# A Lightweight Transformer Edge Intelligence Model for RUL Prediction Classification

**DOI:** 10.3390/s25134224

**Published:** 2025-07-06

**Authors:** Lilu Wang, Yongqi Li, Haiyuan Liu, Taihui Liu

**Affiliations:** College of Computer Science and Technology, Beihua University, Jilin City 132013, China; 13616471056@163.com (L.W.); lyq910241319@163.com (Y.L.); 18052297885@163.com (H.L.)

**Keywords:** lightweight, remaining useful life (RUL) prediction, transformer, BiGRU

## Abstract

Remaining Useful Life (RUL) prediction is a crucial task in predictive maintenance. Currently, gated recurrent networks, hybrid models, and attention-enhanced models used for predictive maintenance face the challenge of balancing prediction accuracy and model lightweighting when extracting complex degradation features. This limitation hinders their deployment on resource-constrained edge devices. To address this issue, we propose TBiGNet, a lightweight Transformer-based classification network model for RUL prediction. TBiGNet features an encoder–decoder architecture that outperforms traditional Transformer models by achieving over 15% higher accuracy while reducing computational load, memory access, and parameter size by more than 98%. In the encoder, we optimize the attention mechanism by integrating the individual linear mappings of queries, keys, and values into an efficient operation, reducing memory access overhead by 60%. Additionally, an adaptive feature pruning module is introduced to dynamically select critical features based on their importance, reducing redundancy and enhancing model accuracy by 6%. The decoder innovatively fuses two different types of features and leverages BiGRU to compensate for the limitations of the attention mechanism in capturing degradation features, resulting in a 7% accuracy improvement. Extensive experiments on the C-MAPSS dataset demonstrate that TBiGNet surpasses existing methods in terms of computational accuracy, model size, and memory access, showcasing significant technical advantages and application potential. Experiments on the C-MPASS dataset show that TBiGNet is superior to the existing methods in terms of calculation accuracy, model size and throughput, showing significant technical advantages and application potential.

## 1. Introduction

The traditional equipment maintenance strategies mainly include passive maintenance and preventive maintenance. These methods wait until the equipment malfunctions before carrying out maintenance, causing production disruptions. Conducting preventive maintenance at fixed intervals may either lead to either excessive maintenance or insufficient maintenance. In contrast, predictive maintenance, by monitoring the status of equipment in real time and predicting potential failures, enables maintenance to be carried out at the optimal time point. This not only avoids the huge losses caused by unexpected shutdowns but also reduces unnecessary maintenance costs. The prediction of the Remaining Useful Life (RUL) of equipment can formulate proactive maintenance plans and reduce the occurrence of safety accidents.

Most of the current mainstream trends in RUL prediction use deep learning models, especially architectures such as recurrent neural networks (RNN), Long Short-Term memory networks (LSTM), and attention mechanisms. They can quickly and effectively capture long-term dependencies in time series data, thereby improving the accuracy of prediction. Yu et al. [1] designed an improved similarity-based remaining useful life prediction algorithm. This algorithm utilizes the bidirectional recurrent neural network (Bi-RNN) autoencoder to enhance the accuracy and robustness of RUL estimation for mechanical systems. In Article [2], Wennian Yu’s method was extensively verified, and it was found that the RNN architecture of the exit control mechanism can better implement the encoding function of time series. In the study, three common RNN architectures (LSTM, PLSTM and GRU) were compared and it was found that their results were similar with no significant differences. Xiang et al. [3] developed a Single Gated recurrent neural network (SGRNNs) and combined it with a differential weighted information storage mechanism for the prediction of the remaining useful life (RUL) of machines. The RNN architecture of the gating mechanism has effective predictive ability.

The network architecture of the gating mechanism can effectively alleviate the problems of vanishing gradient and gradient explosion, thereby better capturing the long-distance dependencies in the sequence and improving the overall performance of the model. Bampoula et al. [4] utilized the deep learning model of Long Short-Term Memory Autoencoders (LSTM Autoencoders) for predictive maintenance. The model was capable of identifying potential faults, thereby reducing unnecessary downtime and lowering maintenance costs. However, the selection of model parameters affects the performance of the network. Increasing the number of layers or neurons has a significant impact on improving the network performance. Ma et al. [5] adopted a new hybrid architecture for the prediction of the remaining useful life (RUL) of rolling bearings. This method combines the multi-scale effective Channel Attention convolutional neural network (MSECNN) and the bidirectional gated recurrent unit (BIGRU), aiming to effectively capture the local and global features in time series data and retain the time dependence, thereby improving the accuracy of RUL prediction for rolling bearings. Sun et al. [6] developed a prediction model based on the CNN-GRU hybrid network in order to solve the problems of insufficient extraction of degraded features, inability to capture long-term dependencies and low prediction accuracy in traditional methods. Compared with the LSTM network, the GRU network provides a simpler solution. However, when dealing with longer sequences, even with the help of a gating mechanism, this type of model must compress the information of the entire sequence into a fixed-size vector (i.e., the final hidden state), which may lead to information loss. Moreover, although it alleviates the problem of vanishing gradients, for particularly long sequences, they still face the problem of information bottlenecks.

The attention mechanism directly extracts the features with higher importance in the original data without relying entirely on the compressed representation of the model, thereby alleviating the problems of network information loss and information bottleneck in the gating mechanism. Wang et al. [7] proposed an adaptive self-attention Long Short-Term memory (SA-LSTM) prediction model for the prediction of the remaining useful life (RUL) of lithium batteries. Combine the attention mechanism with LSTM to adjust the network parameters in real time to adapt to the current local changes. The problem is that the cumulative error increases with the increase in the number of periods, especially when encountering accelerated degradation or local regeneration phenomena, which may cause the predicted value to deviate rapidly from the actual value. Zhu et al. [8] proposed a health index construction method based on residual hybrid networks combined with the self-attention mechanism (Re-HSA), which deeply integrates the attention mechanism, convolutional neural network (CNN), and gated recurrent unit (GRU), and achieves good prediction performance on the test set. Qin et al. [9] developed a new multi head self-attention automatic encoder (SMSAE) method to build health indicators (HI) and predict the remaining service life of machinery (RUL) based on similarity. The multi head attention mechanism is improved and embedded into the automatic encoder to improve the accuracy of RUL prediction. However, in order to achieve higher prediction accuracy, higher computational requirements will be introduced, making it difficult for these models to be deployed to edge hardware, affecting the RUL prediction application in the actual scene. Yu et al. [10] proposed a novel digital twin-driven three-stage feature filling framework named NIFD-Net for non-contact intelligent fault diagnosis. Instead of solely relying on network models for fault diagnosis, this framework establishes a critical relationship between Simulated Vibration Signals (SVS) and Non-Contact Signals (NCS), making it particularly suitable for industrial scenarios where traditional contact-based methods are inapplicable. This approach demonstrates promising potential as a solution for intelligent fault diagnosis in non-contact applications.

This study aims to address three critical issues:

(1) Gated recurrent networks, such as LSTM and GRU, exhibit certain capabilities in processing sequential data of limited length. However, their inherent information compression mechanisms pose significant challenges when handling long sequences. As the sequence length extends, key information may be gradually lost during the iterative updating and filtering operations of the gating units, resulting in poor performance in capturing long-term dependencies. Moreover, these networks lack explicit state classification capabilities, which restricts their effectiveness in tasks that require hierarchical modeling and precise classification of complex sequential states.
(2) CNN–RNN hybrid models integrate the local feature extraction advantages of convolutional neural networks with the temporal dependency modeling ability of recurrent neural networks. While they can effectively extract local features and model time series dynamics, their performance remains constrained by the information bottleneck of the RNN component. The gradient vanishing or explosion issues during the hidden state propagation in RNNs limit the model’s capacity to handle long sequences. Additionally, the intricate architecture of hybrid models, characterized by the interlacing of convolutional and recurrent layers, leads to a large number of parameters. This complexity renders lightweight design extremely challenging, as it is difficult to develop effective strategies for parameter pruning and computational optimization to meet the requirements of resource-constrained environments.
(3) Attention mechanism-enhanced models, such as SA-LSTM and Res-HSA, have significantly improved prediction accuracy by enhancing the model’s ability to focus on key information. Nevertheless, when capturing long-term dependencies, these models face substantial computational burdens. The attention mechanism requires calculating the correlation between all positions in the sequence, resulting in a quadratic increase in computational complexity with the growth of sequence length. This high computational demand leads to slow inference speed and excessive memory consumption, making it difficult to deploy these models on resource-constrained edge devices, where real-time performance and low memory footprint are essential requirements.

These three issues collectively represent the prevalent challenges faced by most existing models. To address these limitations, we propose TBiGNet, a lightweight Transformer-based deep learning model for Remaining Useful Life (RUL) prediction and classification.

In the encoder of TBiGNet, we optimize the Transformer’s attention mechanism to significantly reduce memory access requirements. Additionally, a feature pruning module is incorporated to eliminate irrelevant computations. This module adaptively identifies and discards unimportant features, streamlining the computational process without sacrificing crucial information. In the decoder, to prevent the inadvertent removal of valuable features, we employ Bi-directional gated recurrent units (BiGRU) from gated recurrent networks to fuse the unpruned and pruned features. This feature fusion strategy enhances the model’s stability and accuracy by effectively integrating different levels of feature representations. Finally, a linear prediction layer for RUL is utilized, followed by a threshold-based classification approach to categorize the operational states of machines.

Through the lightweight design of both the encoder and decoder, TBiGNet not only overcomes the issues of high memory consumption of the attention mechanism and difficulties in edge device deployment but also outperforms current state-of-the-art models in terms of parameter count and prediction accuracy, as demonstrated on the C-MAPSS turbofan engine dataset. The experimental results validate the effectiveness and superiority of TBiGNet in practical RUL prediction tasks.

## 2. Related Work

### 2.1. Transformer and GRU

The Transformer model is currently the most widely used and studied neural network model. Proposed by Vaswani et al. [11] in 2017, it has made breakthrough progress in the field of natural language processing with its excellent parallel computing capabilities and advantages in modeling long sequences. Its core attention mechanism can directly model the dependencies between any positions in the sequence. These characteristics make the Transformer architecture highly suitable for handling the timing monitoring data of industrial equipment. However, the existing problems are the insufficiency in extracting complex degradation features from high-dimensional data and the excessive computational load when capturing long-term dependencies. The Gate Recurrent Unit (GRU) [12] was proposed in 2014. Like LSTM, it was put forward to solve the problem of long-term dependence. However, compared with the LSTM network, the GRU network is simpler. It combines the forget gate and input gate in LSTM into one update gate, and has fewer parameters and computational complexity. The bidirectional GRU network can extract richer feature representations, and this ability is conducive to more accurate RUL prediction. Modern RUL prediction often requires processing high-dimensional data from multiple sensors. BiGRU can effectively integrate data from different sources and extract valuable information from them for the RUL prediction of the model, thereby improving the accuracy of the model.

GRU and Transformer can precisely make up for the existing problems of each other. Cao et al. [13] studied a novel framework called VS-TransGRU, which is based on Transformer and GRU (gated recurrent unit) and enhanced through visual-semantic fusion for action prediction from the first-person perspective. Combining the powerful time modeling ability of the Transformer and the flexible iterative characteristics of the GRU, they are, respectively, used as the encoder and decoder to handle problems with different prediction times. Compared with other models, the accuracy has been significantly improved. It indicates that the combination of Transformer and GRU has better performance. Zhang et al. [14] studied a model called Transformer-Encoder-GRU (T-E-GRU), which combines the Transformer Encoder and the gated recurrent unit (GRU) for sentiment analysis of Chinese review texts. The model integrates the powerful global feature extraction ability of the Transformer and the excellent sequence feature extraction ability of the GRU. Not only were the classic recurrent models (such as RNN, LSTM, GRU, etc.) compared, but also the recurrent models with attention mechanisms (RNN-attention, LSTM-attention, etc.). Experiments show that T-E-GRU achieves better results compared with other models, indicating that the combination of the attention mechanism of Transformer with GRU has higher accuracy than that with other cyclic models. However, compared with the LSTM-attention model, the test time required for T-E-GRU on various datasets varies from 6% to 40% higher, indicating the problem of a larger amount of computation. Yan [15] presented a method based on CNN-GRU-MSA (Convolutional Neural Network—gated recurrent unit—Multi-head Self-attention) combined with multi-channel feature fusion for the prediction of the remaining useful life (RUL) of rolling bearings. This model combines the multi-head attention mechanism, convolutional neural network and GRU, further proving that the model combining Transformer and GRU has better performance. Cao et al. [16] proposed a model based on parallel gated recurrent Unit (GRU) and dual attention mechanism to predict the remaining useful life (RUL) of wind turbine bearings. The effectiveness and superiority of the proposed method were verified through the vibration dataset on the PRONOSTIA platform and the dataset of wind turbines in northeastern China. Although the above models have relatively high accuracy, they do not take into account the lightweight design of the models and cannot be deployed on edge devices for real-time RUL prediction.

### 2.2. Lightweight Methods

The application of lightweight models in resource-constrained environments enables real-time Remaining Useful Life (RUL) prediction with low latency. Currently, most models adopt manual pruning to delete irrelevant parameters, reducing model parameter size and alleviating computational load. However, this approach is time-consuming and labor-intensive, hindering rapid deployment, upgrading, and error correction. Ren et al. [17] proposed an edge-intelligent time series reduction network (GT-MRNet) based on lightweight group transformation. The method designs a group linear transformation to reduce Transformer parameters and develops a time series reduction strategy that cuts off unimportant time steps in each layer via adaptive pruning. This strategy minimizes redundant computations by leveraging the importance scores of attention mechanisms. Compared with the standard Transformer, GT-MRNet reduces parameters by up to 74.7% and computational load by 91.8% without accuracy loss, achieving Transformer lightweight design. In [18], a lightweight adaptive knowledge distillation (KD) framework was explored, featuring a multi-head and multi-branch student model for adaptive reasoning on diverse samples. Shi et al. [19] dynamically extracted features by replacing information connections in the gated recurrent unit (GRU) with an adaptive feature extraction operator (Involution), reducing involved parameters. Deng et al. [20] proposed an auxiliary dataset based on deep separable convolution to extract peak values from original vibration signals, lowering computational requirements while enhancing prediction and diagnostic performance of lightweight models. Sun et al. [21] developed a lightweight bidirectional long short-term memory network via adaptive pruning, which identifies and removes redundant elements in the original BiLSTM model, reducing calculation amount by 36% and improving prediction accuracy by 3%. Existing model size and computation reduction methods increasingly trend toward adaptive dynamic pruning strategies. However, such strategies require ultra-highly automated pruning methods tailored to specific models, and controlling their impact on prediction accuracy remains challenging.

### 2.3. Contribution

The main contributions of this article are as follows:

(1) We present TBiGNet, a lightweight Transformer-based architecture. The enhanced encoder–decoder structure significantly boosts the accuracy of the conventional Transformer by over 15%, while also achieving a reduction in more than 98% in both computational load and parameter size.
(2) A novel optimization of the Transformer’s multi-head attention is introduced, where the individual linear mappings of queries, keys, and values are combined into one efficient operation, achieving a 60% reduction in memory access overhead.
(3) A new adaptive feature pruning approach is introduced into the encoder, allowing the model to selectively focus on the most critical features during processing. This strategy helps eliminate unnecessary features and boosts prediction accuracy by 6%.
(4) Feature fusion for two different features is designed in the decoder. Using BiGRU to compensate for the deficiency of the attention mechanism in obtaining degraded features, compared with the traditional Transformer, the computational load and parameter number of the decoder are both reduced by more than 48%, and the model accuracy is improved by 7%.
(5) Extensive experiments were conducted on the C-MAPSS dataset to validate the effectiveness of the proposed model. The results clearly show that TBiGNet surpasses existing methods in calculation accuracy, model size, and computational efficiency.

## 3. Methods

The TBiGNet model follows the overall architecture of the Transformer model and uses a stacked encoder with feature clipping module, efficient multi head attention mechanism and feedforward network. The original Transformer stacking method is abandoned in the decoder and multi-scale feature fusion is used to output the results.

### 3.1. Overall Framework

**Figure 1:**
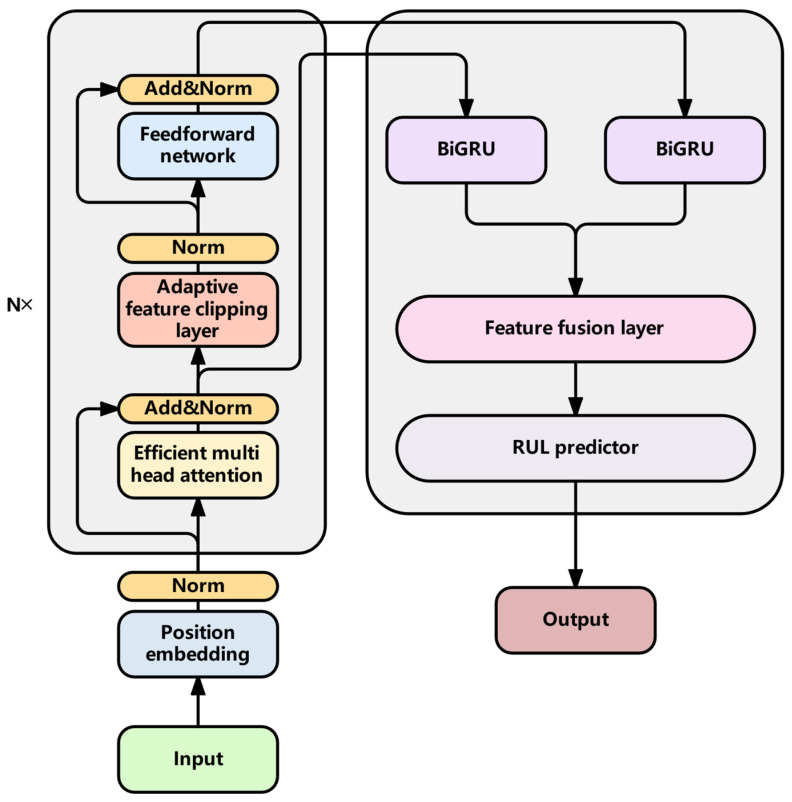
Architecture diagram of Model TBiGNet.

The model framework is shown in Figure 1. The gray part on the left is the encoder and the gray part on the right is the decoder. After preprocessing, the data first passes through the encoder module. The data is stacked through multiple encoder modules to extract and crop features. Each module contains an improved efficient multi-head attention mechanism, an adaptive feature cropping layer, and a feedforward network. Moreover, the number of feature cropping increases with the increase in layers. Specifically, the more layers are stacked, the more features are cropped. After encoding, two sets of features are obtained: one is the original feature and the other is the trimmed feature. These two groups of features are then input into the Decoder module. The original features are extracted from the attention of the first encoder module and then input into the decoder, with the aim of preventing useful features from being cropped out. In order to effectively integrate the original features and crop the feature information, and at the same time utilize the bidirectional information flow of BiGRU to enhance the understanding ability and expressiveness of the model, the two groups of features are, respectively, processed by two independent BiGRU networks, and then the processed features are fused. Finally, the features are linearly predicted for RUL (Remaining Useful Life) through the RUL predictor module. The results of the linear prediction are then binary classified through threshold classification to determine whether the equipment is in a maintenance state (RUL ≤ 30) or a normal operating state (RUL > 30). The entire process outputs the predicted RUL value and the corresponding classification probability.

### 3.2. Encoder Module

The traditional Transformer encoder has a large amount of memory access and many parameters. Therefore, it was modified on this basis. First is the efficient multi-head attention mechanism module. It only reads the input data once and, after passing through a linear layer, splits to generate Q, K, and V, reducing the model’s memory access by more than 60%. Then, the initial feature clipping is carried out through the adaptive clipping layer. The clipping ratio is controlled by parameters. The deeper the layer, the more clipping is performed to reduce the computational load for the subsequent decoder module. However, at the same time, a limit on the minimum number of retained features is set to ensure that the information is not overly lost. Finally, after the simplified feedforward network processing, the number of parameters is reduced by more than 40%. The encoder module is a sequence of modules composed of multiple encoder layers stacked together. The internal structure of each stacked layer is the same, all including an efficient multi-head attention layer, an adaptive clipping layer and a feedforward network layer.

#### 3.2.1. Efficient Multi-Head Attention Mechanism

**Figure 2:**
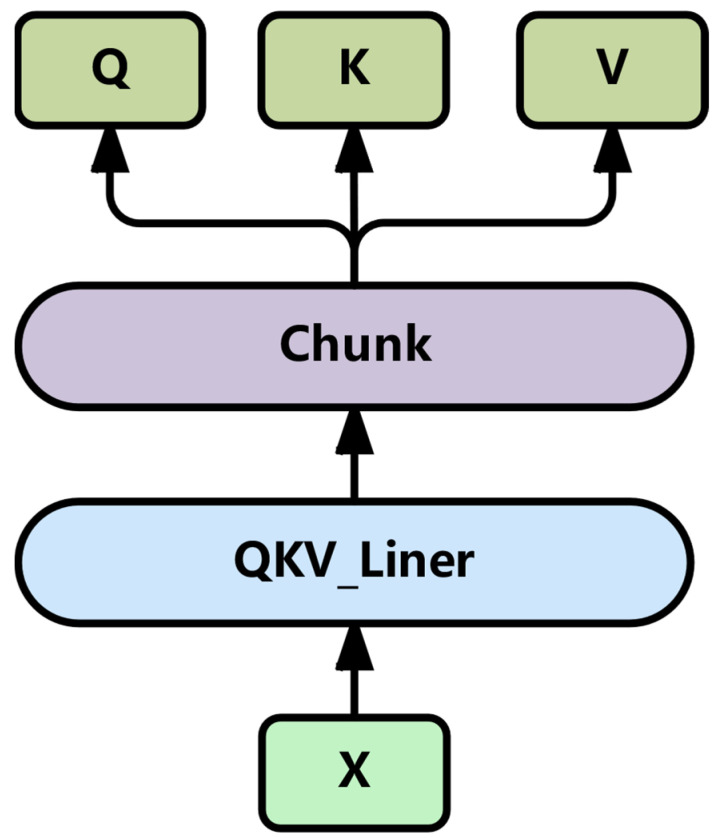
Generation of Q, K, and V matrices.

The traditional multi-head attention mechanism of Transformer uses three linear layers to read the input data, respectively, to obtain three matrices Q, K, and V. In an environment with limited computing resources, multiple memory accesses are required when inputting complex degraded data, which will cause an increase in hardware resource consumption and latency. To address this issue, this paper improves an efficient multi-head attention mechanism based on the traditional Transformer multi-head attention mechanism. Firstly, a linear layer and segmentation method were used to replace the original three linear layers to generate the values of Q, K, and V, reducing the memory access volume by more than 60%. The specific method is shown in Figure 2.

Specifically, a single linear layer is used to map the input data to three dimensions of hidden layer sizes (which contain information about queries, keys, and values), and then it is divided into three parts, corresponding to queries, keys, and values, respectively. This method not only reduces the memory access volume, but also can capture the information in different subspaces, maintaining the advantages brought by the multi-head attention mechanism.

**Figure 3:**
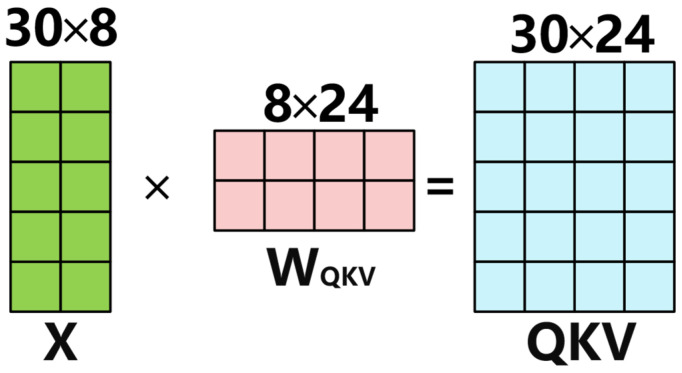
The input data passes through a weight matrix.

Assuming the hidden dimension is 8, the sequence length is 30, and the number of attention heads is set to 8, the input data passes through the first weight matrix as shown in Figure 3. First, the input X undergoes a linear transformation to obtain a matrix-vector of Query, Key, and Value, as described in Equation (1).

(1) $$QKV={XW}_{QKV}$$

Here, $X\in R^{B\times S\times d_{model}}$ represents the position-encoded input tensor, where B denotes the batch size, S is the sequence length, and $d_{\mathrm{model}\;}$ is the hidden layer dimension of the model. The weight matrix $W_{QKV}\in R^{d_{model}\times 3d_{model}}$ is designed to map the input into a combined space of Query, Key, and Value, thereby generating the QKV tuple.

In traditional attention mechanisms, the input data $X\in R^{B\times S\times d_{model}}$ requires three accesses, while the weight matrices $W_{Q},W_{K},W_{V},W_{O}\in R^{d_{model}\times d_{model}}$ necessitate four accesses. In contrast, the proposed efficient attention mechanism only accesses the input data $X\in R^{B\times S\times d_{model}}$ once and the weight matrices twice: the first access involves the input weight matrix $W_{QKV}\in R^{d_{model}\times 3d_{model}}$, and the second access targets the output weight matrix $W_{o}\in R^{d_{model}\times d_{model}}$. Although the weight matrices differ, both attention mechanisms incur the same memory access for computing other results, amounting to $4d_{model}^{2}$. The key differentiator lies in the memory access of input data: traditional attention requires $3\times (B\times S\times d_{model})$ accesses, whereas the efficient attention reduces this to $B\times S\times d_{model}$, resulting in a 60% decrease in memory access for input data. This significant reduction endows the efficient attention mechanism with a distinct advantage when deployed on resource-constrained edge devices.

**Figure 4:**
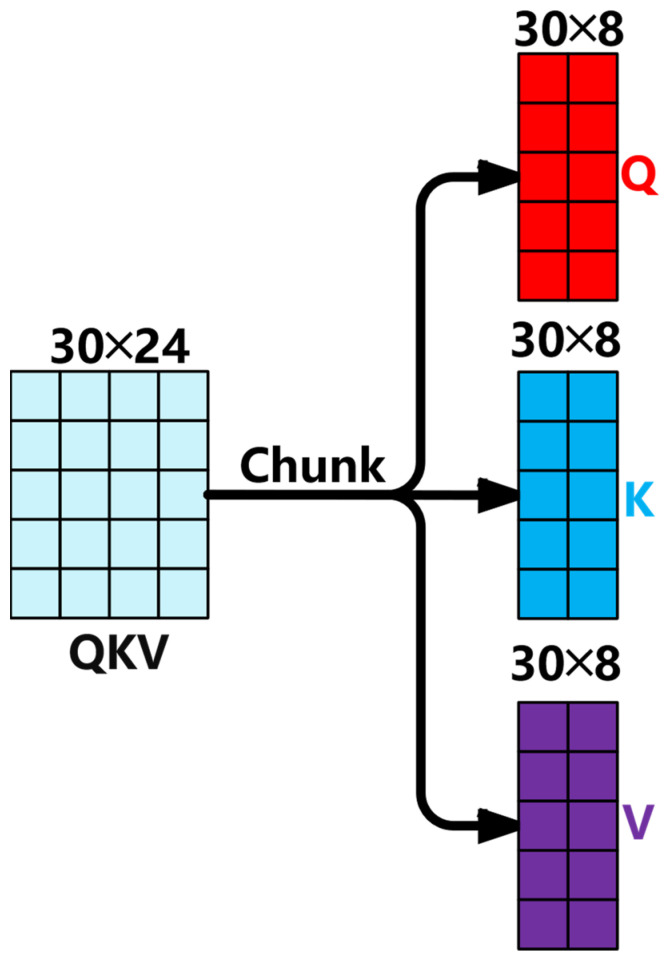
Splitting of the Q, K, and V matrices.

The configuration of the Q, K, and V matrices based on the model parameters mentioned above is shown in Figure 4. The QKV tuple is divided into three parts: Q (query vector), K (key vector), and V (value vector), as shown in Formula (2).

(2) [Q,K,V]=chunk(QKV)

**Figure 5:**
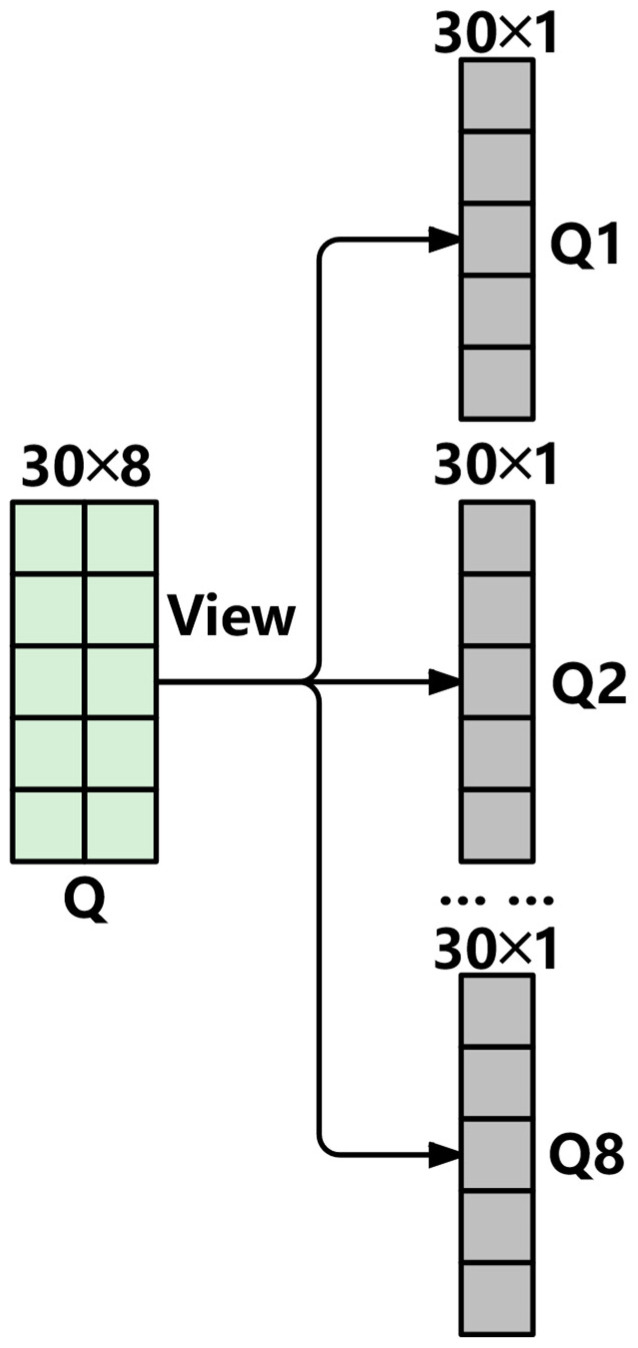
Division of the attention head matrix.

$Q,K,V\in R^{B\times S\times d_{model}}$ is the query vector, key vector and value vector. Then, perform the Q, K, and V matrix division for each attention head. For example, the Q matrix division is shown in Figure 5. The original hidden dimension $d_{model}$ is reorganized into the form of $H\times d_{head}$, where H is the number of attention heads and $d_{head}$ is the dimension of each head. The divided dimensions are $Q_{h},K_{h},V_{h}\in R^{B\times H\times S\times d_{head}}$, where h represents the serial number of the attention head. The purpose is to enable each attention head to handle each element in the sequence independently, so that the attention scores and context vectors of all heads can be calculated in parallel.

(3) $$\mathrm{Softmax}(x)==\frac{e^{z_{i}}}{\sum_{i=1}^{N}e^{z_{i}}}$$

Formula (3) is the Softmax function. Here, $z_{i}$ represents the i-th element of vector Z, and N is the total number of elements.

Formula (4) is used for calculating the attention score.

(4) $$Attention(Q_{h},K_{h},V_{h})=softmax\left(\frac{Q_{h}K_{h}^{T}}{\sqrt{d_{h}}}\right)V_{h}$$

$d_{h}$ is the scaling factor and the scaling operation is performed. Among them, $Q_{h},K_{h}$ and $V_{h}$ are obtained from the above text. After the attention score is calculated, the outputs of all heads are merged back to the original dimension. The specific method is shown in Formula (5).

(5) $$MultiHead(Q,K,V)=Concat({head}_{1},\ldots ,{head}_{h})W^{O}$$

(6) $$head_{i}=Attention(Q_{i},K_{i},V_{i})$$

In Formula (5), $head_{i}\in R^{B\times S\times d_{head}}$ is the output of the h head, and $W^{O}\in R^{d_{model}\times d_{model}}$ is the weight matrix of the output linear transformation.

#### 3.2.2. Adaptive Feature Cropping Layer

**Figure 6:**
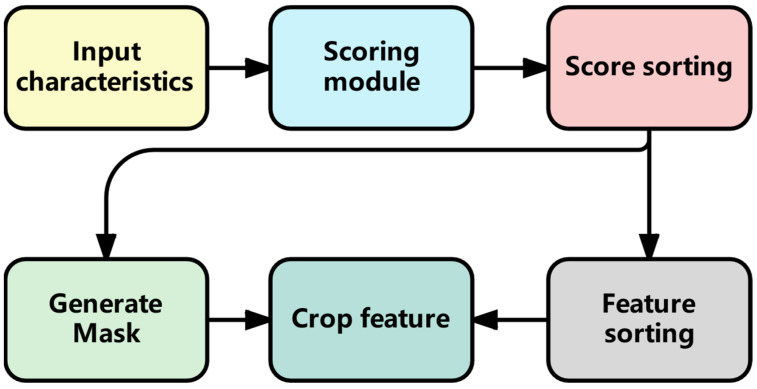
Adaptive feature clipping process.

The adaptive clipping layer first evaluates the importance of features through an importance scoring module, dynamically determines the clipping threshold based on the predicted importance score, uses the clipping threshold to determine the clipping mask, calculates the input features and the mask, and finally outputs the clipping features. This adaptive clipping strategy can reduce the influence of redundant features. The specific cropping process is shown in Figure 6.

The method of the feature importance scoring module is shown as Formula (7).

(7) $$I(x)=\sigma (W_{2}\cdot ReLU(W_{1}x+b_{1})+b_{2}))$$

Among them, $x\in R^{B\times S\times d_{model}}$ is the input feature, $W_{1}$, $W_{2}$ are the weights of the linear layer, $b_{1}$, $b_{2}$ are the bias terms, σ is the sigmoid function, and ReLU is the activation function. Firstly, the input feature x passes through the first linear layer $W_{1}x+b_{1}$, and then undergoes nonlinear transformation through the ReLU activation function to eliminate negative values. Then, this result passes through a second linear layer $W_{2}$ and bias $b_{2}$. Finally, the sigmoid function σ is used to compress the output to the interval of [0, 1] to obtain the importance score of each feature. The closer this score is to 1, the more important this feature is. The closer it is to 0, the less important the feature is. Among them, the weight matrix can be adjusted in a timely manner along with the loss function during training to adapt to different input features and dynamically identify important features.

In the first linear layer, the weight matrix $W_{1}\in R^{{d_{model}\times (d}_{model}/2)}$ and the bias vector $b_{1}\in R^{{(d}_{model}/2)}$ are responsible for learning the complex relationships among features. In order to capture the relationship between different features, the dimension of the feature is compressed to half of it, so that the key feature information can be extracted and the correlation between the input features can be learned. The weight matrix $W_{2}\in R^{{(d}_{model}/2)\times 1}$ and the bias vector $b_{2}\in R^{1}$ in the second linear layer are responsible for making the final judgment, aggregating the intermediate features processed by the first linear layer into a single score. Each element of W_2_ corresponds to the importance weight of the intermediate feature, and finally outputs a score value representing the importance at a certain time step. Formulas (7)–(10) are all used to determine the calculation of the mask value. The obtained mask is subjected to element-level product calculation with the input feature x, redundant features are trimmed off, and the trimmed features are output. The entire process can be regarded as a scorer of feature importance, learning the importance of features through multiple layers of transformation. Then, sorting and threshold selection are carried out. The specific calculation formulas are shown in (8), (9), and (10).

(8) sorted,indices=sort(I(x))

(9) $$x_{sorted}=x[indices]$$

(10) threshold=sorted[K−1]

I(x) is the feature importance score, sorted is the sorted importance score, indices is the sorted index, which is used to sort the features, sort is the descending sort, and $x_{sorted}$ is the features resorted according to the index. The arrangement order of the features corresponds one-to-one with their own importance score positions. $k_{t}$ is the number of retained features dynamically calculated based on the current layer position. Since the index starts from 0, the index of the K-th value is K − 1, which is used to determine the threshold. The specific calculation methods are shown in Formulas (13) and (14). threshold is a threshold value, serving as the dividing line for feature clipping and providing a basis for subsequent mask generation. First of all, calculate the importance score of each feature through the importance prediction module $I\left(x\right)$. Then, sort these scores and features in descending order, and select the importance score of the K-th feature as the threshold. Next, compare all the importance scores with this threshold to generate a binary mask.

(11) mask=sorted≥threshold

Formula (11) is the mask generation method. Positions greater than or equal to the threshold are 1, and positions less than the threshold are 0.

(12) $$x_{pruned}=x_{sorted}⊙mask$$

Finally, the Hadamard product (⊙) of the original feature and this mask is performed using Formula (12) to achieve the selective retention of features. Features below the threshold will be set to zero, thereby achieving the purpose of feature clipping. The calculation method of $k_{t}$ is as follows:

(13) $${pruning}_{size}={initial}_{K}*({decay}_{rate}{)}^{{layer}_{idx}}$$

(14) $$K=max({pruning}_{size},\frac{{initial}_{K}}{4})$$

${pruning}_{size}$ is the quantity to be trimmed and retained, which is calculated by Formula (13). K is the number of retained features in the current layer, ${initial}_{K}$ is the initial number of retained features, ${decay}_{rate}$ is the decay rate, and ${layer}_{idx}$ is the index of the current layer. This formula calculates the number of features that need to be retained in each layer. First of all, ${initial}_{K}$ is the initial number of features, ${decay}_{rate}$ is the decay rate, and ${layer}_{idx}$ is the index of the current layer (starting from 0). Formula (13) multiplies the initial number of features by the current layer as a power-exponential attenuation rate. In this way, as the number of layers increases, the number of retained features will gradually decrease. For example, the initial number of retained features is set to 26 features; that is, the initial layer has 26 features, and the attenuation rate is set to 0.9. The first layer is 26 × 0.9, retaining 23 features, and the second layer is 26 × 0.9^2^, retaining 21 features.

Formula (14) sets a restriction on the minimum number of retained features, which should not be less than ${initial}_{K}$/4 (e.g., 26/4≈6). This is designed to prevent excessive feature pruning. In multiple time steps, there exist numerous repeated and redundant features. Too few features may lead to model underfitting, while excessive features might cause overfitting. Based on empirical values, the minimum number of retained features for the K value is set to be no less than one-fourth. Such a design ensures a gradual reduction in the number of features while maintaining necessary information content, see Algorithm 1.
**Algorithm 1** Feature Clipping Step**Input:** The extracted feature X, attenuation rate ${layer}_{idx}$, initial reserved feature number ${initial}_{K}$, encoder layer number N
**Output:** The fully cropped feature $X_{Prun\mathrm{e}d}^{N}$
1: **for** i ← 1 to N **do**
2:  The feature X_i_ extracted by the layer i encoder is scored by the Formula (7) based on the importance scoring module
3:  Calculate the number of features K retained in the current layer based on Equations (13) and (14)
4:  Sort the importance scores and features in descending order and calculate the threshold by Equations (8)–(10)
5:  Generate the binary mask using Equation (11)
6:  The input feature X_i_ is calculated by Formula (12) and the mask to obtain the feature $X_{Prun\mathrm{e}d}^{i}$ trimmed by the ith layer encoder
7:  **end for**
8: **return** Fully cropped feature $X_{Prun\mathrm{e}d}^{N}$


The design of the whole encoder reflects the idea of “progressive feature extraction”. From the shallow layer, more features are retained to the deep layer, which not only ensures the full extraction of information, but also realizes the cutting and optimization of features. At the same time, the dual information flow is composed of the original features and the encoded features, which provides a rich feature representation for the subsequent decoder module.

#### 3.2.3. Feedforward Network Layer

The feedforward network layer consists of two linear transformations, along with a ReLU activation function and a Dropout function. According to the statistical information in article [22], the expansion ratio of the middle layer is determined according to the different functions implemented by different models, and the expansion ratio of the middle layer of the feedforward network of most models is set to 4 times. In the traditional Transformer feedforward network, the dimension of the middle layer is extended by 4 times. The feedforward network in the proposed model is expanded by 2 times, which aims to realize the lightweight of the model, reducing more than 40% of the parameters and calculation of the traditional Transformer feedforward network. The specific method is shown in Equation (15).

(15) $$FFN(x)=Dropout(W_{2}\cdot (Dropout(ReLU(W_{1}\cdot X+b_{1})))+b_{2})$$

The input characteristics are marked as X, W_1_ and W_2_ respectively represent the weight parameters of the first and secondary linear mapping, while b_1_ and b_2_ are the corresponding offset parameters. The first linear layer $W_{1}\in R^{d_{ff}\times d_{model}}$ bias vector $b_{1}\in R^{d_{ff}}$ is used to expand the hidden dimension $d_{model}$ by a factor of 2, that is, $d_{ff}=2\times d_{model}$. The dimension extension of W_1_ increases the expression ability of the model, where $d_{ff}$ represents the middle layer dimension of the feedforward network, and $d_{model}$ represents the hidden dimension set in advance. Activation layer ReLU introduces nonlinearity and prevents vanishing gradients. The second linear layer $W_{2}\in R^{d_{model}\times d_{ff}}$ bias vector $b_{2}\in R^{d_{model}}$ is used to compress the feature from 2 times of $d_{model}$ back to the original $d_{model}$ and restore the feature dimension. The Dropout function is used to prevent over fitting.

### 3.3. DecoderModule

The Decoder layer mainly processes two types of inputs: the original features and the features trimmed by the encoder. Firstly, the transformed original features and the cropped features are processed, respectively, by two independent BiGRU networks. Then, the results of the two are concatenated, and finally RUL prediction is carried out.

#### 3.3.1. BiGRU

**Figure 7:**
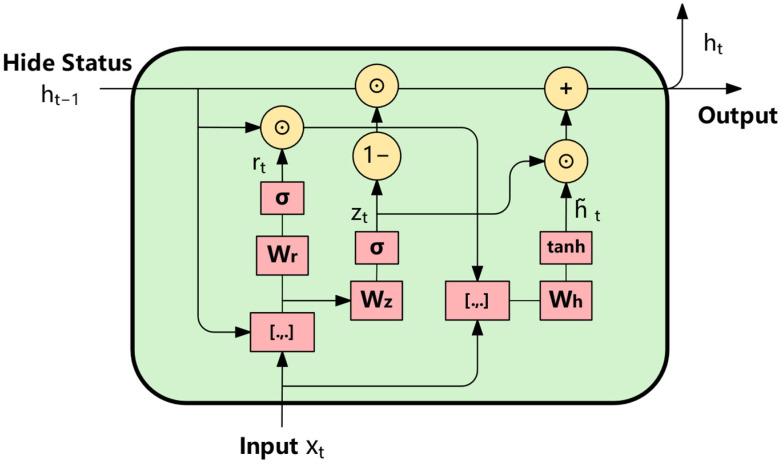
GRU unit.

GRU has the characteristics of less computation and fewer parameters, and can also alleviate the problem of gradient disappearance. It realizes the dynamic filtering and updating of information and reduces the complexity of the model. Because of its simple structure, GRU can train faster. Among them, BiGRU (Bidirectional Gated Loop Unit) can obtain the global dependencies in the data sequence. The internal structure of GRU is shown in Figure 7.

First, the input sequence is ${X=(x}_{1},x_{2},x_{3},\ldots {{,x}_{t},\ldots ,x}_{n})$, where $x_{t}\in R$, the time step is t ∈ [1,n], and n represents the length of the sliding window.

Specifically, the gating mechanism in the forward GRU can be expressed by the following formula [23]:

(16) $$z_{t}=\sigma (W_{z}\cdot [h_{t-1}{,x}_{t}])$$

(17) $$r_{t}=\sigma (W_{r}\cdot [{h_{t-1},x}_{t}])$$

(18) $${\tilde{h}}_{t}=tanh(W_{h}\cdot [r_{t}⊙h_{t-1}{,x}_{t}])$$

(19) $$h_{t}=(1-z_{t})⊙h_{t-1}+z_{t}⊙{\tilde{h}}_{t}$$

In the time step *t*, the output of GRU is represented by the current hidden state $h_{t}$, while $h_{t-1}$ represents the hidden state at the previous time. The update gate controls which parts of the memory in the previous time step need to be retained and transferred to the current state, and also determines how much information in the current input should be included in the new memory state, further affecting the fusion ratio between the new and old states. Its calculation method is shown in Formula (16). The reset gate is used to control how much content in the previous memory should be ignored or reset, so as to help the model integrate the new information currently input more effectively. See Formula (17) for the specific expression. The input feature is represented as $x_{t}$, and the symbol ⊙ represents Hadamard product (that is, multiply by element). Candidate hiding states ${\tilde{h}}_{t}$ are generated by the information adjusted by the reset gate, and finally combined with the update gate to determine the hiding state at the current time. The calculation formula of the candidate state is shown in (18). The function *σ* represents the sigmoid activation function, which is used to map the value to the [0, 1] interval; And tanh is a hyperbolic tangent function, which is used to compress data to the range of [−1, 1]. The parameters $W_{z}$, $W_{r}$ and $W_{h},$ respectively, represent the releasable weight matrix corresponding to the update gate, reset gate and candidate status. Finally, all the above intermediate variables will be substituted into Formula (19) to calculate the hidden state of the current time step.

**Figure 8:**
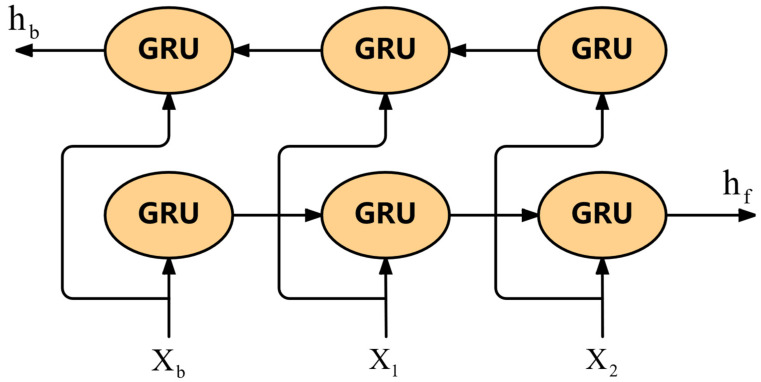
Biru model architecture diagram.

Birgus is to splice multiple GRU to form a two-way GRU network, as shown in Figure 8. The forward propagating GRU and the reverse propagating GRU are calculated by Formulas (20) and (21).

(20) $$h_{f}=GRU(h_{t-1},x_{t})$$

(21) $$h_{b}=GRU(h_{t+1},x_{t})$$

In the end, bigram simply concatenates the forward and backward outputs.

(22) $$h_{t}=[h_{f};h_{b}]$$

For each time step, the two kinds of features processed by BiGRU are concatenated along the last dimension, as shown in Equation (22).

#### 3.3.2. Feature Fusion Layer

**Figure 9:**
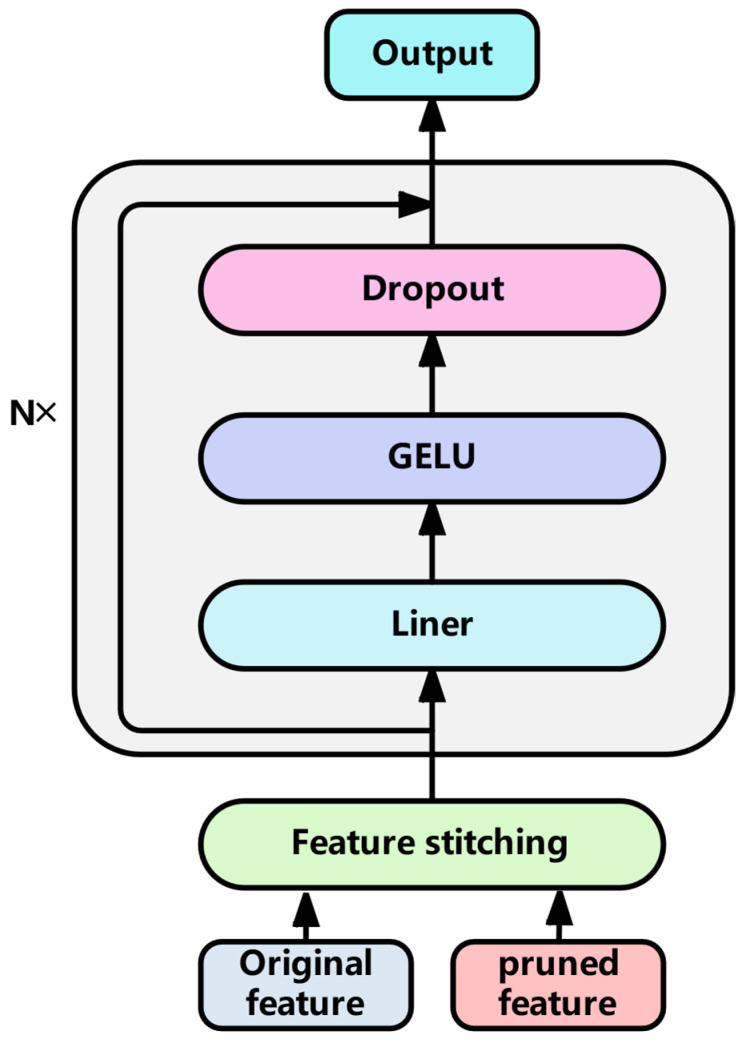
Feature fusion layer.

The concatenated features go through one or more nonlinear transformation layers, that is, a sequence network containing GELU activation function, Dropout, and linear layers. Adding residual connections after each layer ensures that the gradient can be passed back to the previous layers more directly, as shown in Figure 9, which helps to train deeper network structures.

The specific feature fusion algorithm is as follows:

(23) $$F_{0}=[F_{orig};F_{pruned}]$$

(24) $$F^{\prime }=Dropout(GELU\left(W_{k}\cdot F_{k-1}+b_{k}\right)$$

(25) $$F_{k}=F_{k-1}+F^{\prime }$$

Formula (23) is used for the concatenation calculation of original features and trimmed features, and $F_{0}$ is the concatenation result. Where $F_{orig}$ is the original feature, $F_{pruned}$ is the pruned feature, $W_{k}\in R^{d_{model}\times d_{model}}$ and $b_{k}\in R^{d_{model}}$ are the weight matrix and bias vector of this layer, respectively. The $W_{k}$ weight matrix is used to fuse the original feature and the pruned feature. Feature expression is enhanced by linear and nonlinear transformations, and multi-layer stacking allows more complex feature interactions. Where $d_{model}$ is the hidden dimension, k represents the number of layers of the current feature fusion layer, and k is calculated from 1. $F_{k}$ is the output of the K layer of the decoder stack. $F_{k-1}$ is the input feature of the current layer. GELU is the activation function. Dropout is a technique used to prevent overfitting. $F^{\prime }$ is the intermediate feature of the current layer, as shown in Equation (24). After each layer processing, $F_{k-1}$ is connected with $F^{\prime }$ to calculate the residual to obtain the output result $F_{k}$ of the current layer, as shown in Equation (25). If only one feature fusion layer is stacked, then $F_{k}$ is directly transmitted to the RUL predictor. If there are multiple such layers, the above process of nonlinear transformation and residual connection is repeated, that is, Equations (24) and (25) continue to be computed until the last layer, where each layer learns a richer feature representation based on the previous layer, see Algorithm 2.
**Algorithm 2** Multi-Scale Feature Fusion**Input:** Original feature $F_{orig}$, fully trimmed feature $F_{pruned}$, stacked feature fusion layers N
**Output:** Characteristic of the last time step $F_{N}^{N}$
1:$F_{orig}$ and $F_{pruned}$ use Formula (23) to splice features to obtain $F_{0}$
2: **for** i ← 1 to N **do**
3:   Input $F_{i-1}$ into Formula (24) to obtain the intermediate feature $F^{\prime }$ of the current layer
4:   $F_{i-1}$ and $F^{\prime }$ are calculated through the residual link of Formula (25) to obtain $F_{i}^{i}$
5: **end for**
6: **return**  Characteristic of the last time step $F_{N}^{N}$


The RUL predictor is mainly responsible for converting the features obtained after the feature fusion layer processing into the final RUL (Remaining useful life) prediction value. The RUL predictor is calculated as shown in Equation (26).

(26) $$RUL_{pred}=W_{2}\cdot ReLU(W_{1}\cdot X+b_{1})+b_{2}$$

X represents the feature of the last time step of the feature fusion layer, $W_{1}\in R^{d_{model}\times {(d}_{model}/2)}$, $W_{2}\in R^{{(d}_{model}/2)\times 1}$ is the weight matrix, $b_{1}\in R^{d_{model}/2}$, $b_{2}\in R^{1}$ is the bias term. ReLU is the activation function. $W_{1}$ realizes the feature dimension reduction and extracts the key features related to RUL prediction, and $W_{2}$ maps the features to the RUL prediction values to obtain the final prediction results.

## 4. Experiment

In the experiment, 14 key sensor features in the C-MAPSS dataset are used to smooth the data exponentially to reduce noise. Time series characteristics are obtained by the sliding window, and statistical characteristics (mean, standard deviation, etc.) are calculated. For samples close to fault (RUL ≤ 30), Gaussian noise and time warping are used for data enhancement to balance data distribution.

In the model training phase, the learning rate scheduler with attenuation and the AdamW optimizer are used for training. Weighted random sampler is used to deal with the problem of category imbalance. During the training, the early stop strategy was used, and the model parameters of the best RMSE indicators on the validation set were saved.

In the outcome evaluation phase, the model is comprehensively evaluated, including regression and classification indicators. At the same time, the model complexity is analyzed. Finally, the prediction results are displayed by visualization, including the RMSE and confusion matrix of RUL prediction, and all evaluation results are saved to the experiment directory. The experimental environment is Python 3.12.4 equipped with PyTorch 2.5.1+cu124, using uses the 13th Gen Intel(R) Core(TM) i9-13900HX 2.20GHz processor (Intel, Santa Clara, CA, USA) and NVIDIA(R) GeForce RTX(TM) 4080 Laptop GPU (NVIDIA, Santa Clara, CA, USA).

### 4.1. Dataset Preprocessing

**Figure 10:**
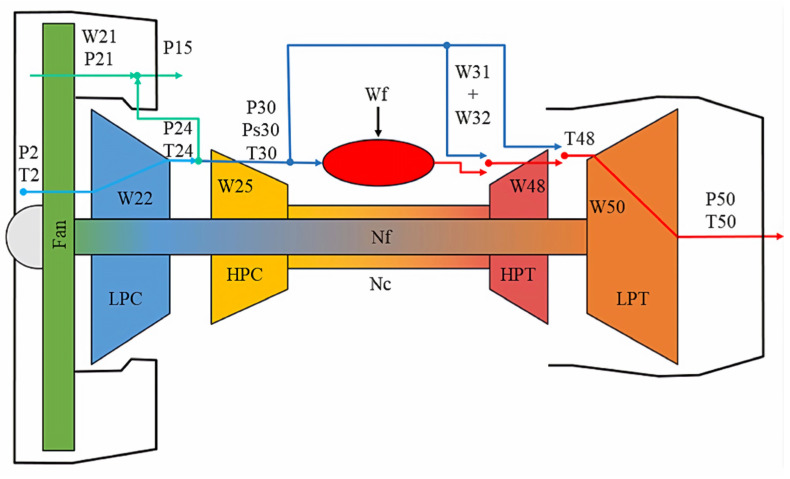
Schematic diagram of the C-MAPSS model [24].

In this study, the publicly available turbofan engine degradation monitoring dataset provided by NASA was used. The dataset includes key engine components, and its structural layout is shown in Figure 10. It consists of four different sub datasets, each representing different operation scenarios and fault conditions. In addition, each sub dataset is divided into training and testing subsets. The training data captures multiple states in the whole degradation process from normal operation to failure, while the test data includes the measurements made at a specific time before the failure and the corresponding residual service life (RUL) values.

#### 4.1.1. Sensor Signal Selection

Each data subset is presented in Table 1. First, 14 key sensor features were selected from the 21 sensor signals (Lines 6–26), and exponential smoothing was applied to each sensor data to reduce noise. This is because although the dataset contains 21 sensor signals, not all sensors provide informative data—some yield constant or discrete values. Thus, we adopted the 14 sensors recommended by Zhang et al. [25] and Li et al. [26] for analysis. The data were then converted to 64-bit floating-point types, with each sensor feature undergoing exponential smoothing and standardization to achieve a mean of 0 and standard deviation of 1. Meanwhile, Remaining Useful Life (RUL) values were clipped within the range of 0 to 125 to avoid an excessively large prediction scope. The detailed information of the four subsets is shown in Table 2.

#### 4.1.2. Exponential Smoothing

Exponentially smoothed using the method of exponentially weighted moving average (EWMA), as shown in Formula (27).

(27) $$S_{t}=\alpha \cdot x_{t}+(1-\alpha )\cdot S_{t-1}$$

$S_{t}$ is the smoothing value at time t, α is the smoothing factor, and $x_{t}$ is the true value at time t.

#### 4.1.3. Standardized Treatment

Since there are large differences among the signal data, it is necessary to standardize the data. Z-Core standardization is used, as shown in Formula (28).

(28) $$Z=\frac{x-\mu }{\sigma }$$

The standardized value Z is computed by transforming the original data X, where μ is the mean and σ is the standard deviation estimated from the training data.

#### 4.1.4. Sample Construction

A common sliding window approach [27] was employed, with the window size set to 30 and the sampling data stride set to 1. The training set was divided into a training subset and a validation subset at an 8:2 ratio. For each device unit in the test set, the same 14 sensor features were used, along with the same exponential smoothing and standardization processing. If the sequence length of a device was insufficient for the window size, zero padding was performed at the front of the sequence to achieve the required length. The label for each window was taken as the RUL value at the last moment of the window. Finally, the labels for the test set were obtained from a separate RUL file. Additionally, the RUL values of both the training set and the test set were constrained within the range of 0–125. All processed data were saved in NumPy array format to facilitate subsequent training.

### 4.2. Training Methods and Evaluation Indicators

The training of the model uses a variety of strategies to improve the performance. First, the AdamW optimizer is used, combined with the weight attenuation of 0.0001 to prevent over fitting. CosineAnnexingWarmRestarts is used for learning rate scheduling. In the initial configuration, the learning rate is set to 0.001. It restarts every five cycles. The minimum learning rate is 0.00001. To mitigate the effects of imbalanced data distribution, WeightedRandomSampler is used to conduct weighted sampling on samples, so that samples in maintenance status (RUL ≤ 30) and normal operation status (RUL > 30) can be balanced in training. We set the training batch size to 32, employ a Dropout value of 0.4, and use 8 attention heads. The model is trained for up to 150 epochs, with early stopping utilized to optimize convergence. When the verification loss does not improve in 15 epochs, the training is stopped. The model stacks two encoder modules, and the feature fusion layer in the decoder stacks two. Each sub dataset is trained 10 times, and the best result is selected as the final result.

The classification of equipment status in TBiGNet is achieved by applying predefined thresholds to the output of the RUL predictor, as shown in Equation (29). Specifically, a threshold of 30 is used to divide the equipment status into two categories: maintenance status (RUL ≤ 30) and normal operation status (RUL > 30). It should be noted that this threshold is set empirically to demonstrate the effectiveness of the model, and the specific threshold can be flexibly adjusted according to different application scenarios.

This threshold-based classification method serves a dual purpose: it not only provides a practical approach to interpreting RUL predictions but also acts as a verification indicator for model accuracy. Regardless of the threshold setting, TBiGNet consistently demonstrates superior performance in correctly classifying equipment status, highlighting the model’s robustness and reliability. This provides effective data support for equipment maintenance staff, facilitating efficient equipment maintenance management.

(29) $$class=\left\{\begin{array}{c} 1\;\;\;\;\;\;\;\;RUL_{pred}>30\\ 0\;\;\;\;\;\;\;\;RUL_{pred}\;\leq 30\;\;\;\; \end{array}\right.$$

$RUL_{pred}$ is the RUL predicted value. Class is the classification result.

Calculate the regression task predicted by RUL during training. For regression tasks, use the RMSE (root mean square error) loss function, as shown in Formula (30).

(30) $$RMSE=\sqrt{\frac{1}{N}\sum_{i=1}^{N}{\left({\hat{y}}_{i}-y_{i}\right)}^{2}}$$

n represents the total number of samples, $y_{i}$ represents the true label of the ith sample, and ${\hat{y}}_{i}$ represents the predicted output of the corresponding sample.

Evaluation indicators include regression indicators and classification indicators. Regression indicators include RMSE (root mean square error) and scoring function. The scoring function is shown in Formula (31).

(31) $$Score=\left\{\begin{array}{l} \sum_{i=1}^{N}\left(e^{-\frac{{\hat{y}}_{i}-y_{i}}{10}}\right)-1,\;\;{\hat{y}}_{i}-y_{i}<0\\ \sum_{i=1}^{N}\left(e^{\frac{{\hat{y}}_{i}-y_{i}}{13}}\right)-1,\;\;{\hat{y}}_{i}-y_{i}\geq 0 \end{array}\right.$$

Here, N refers to the sample size, $y_{i}$ refers to the true target value of the ith sample, and ${\hat{y}}_{i}$ refers to the value predicted by the model.

The classification index is the accuracy rate, as shown in Formula (32).

(32) $$Accuracy=\frac{TP+TN}{TP+FP+FN+TN}$$

TP represents the number of samples that are actually positive and correctly identified as positive by the model; TN represents the number of samples that are actually negative and are accurately predicted to be negative; FP refers to the situation where the model mistakenly judges a negative class as a positive class; FN represents the number of samples that are actually positive but are incorrectly predicted to be negative.

In addition, the visual evaluation of classification is also carried out through confusion matrix. The complexity evaluation of the model includes FLOPs calculation, parameter statistics and model size analysis.

### 4.3. Comparative Experiment

In order to prove the superiority of this method, we compared it with several new methods. The regression evaluation indicators of each method are listed in the following table:

Table 3 summarizes our methods and SVR [28], CNN [29], DBN [25], ELM [25], GB [25], RF [25], LSTM-FNN [30], GAN [31], IDMFFN [32], BIGRU-TSAM [33], AM-LSTM [34], AGCNN [35], GCU-Transformer [36], Cau-AttnPINN [37], BTCAN [38], PAOLTransformer [39] for comparison between methods, the best result has been bold and black. Since the model design is to simulate the scene with limited computing resources, there is a large room for improvement in the evaluation indicators. As shown in Table 3, the method in this paper is superior to the comparison method in all evaluation indicators in FD002 and FD004 datasets. The optimal results in Table 3 have been highlighted in bold.

Since few studies provide references for relevant computational metrics, only six models were selected for comparison, with the best results bolded. As shown in Table 4, both the parameter number and FLOPs of our method demonstrate significant improvements over existing approaches. The model achieves 131.59 K FLOPs (floating-point operations per second) and contains only 1.89 K parameters, featuring a compact size and low computational complexity. These advantages enable it to better adapt to various edge devices and lightweight computing scenarios, meeting the needs of intelligent industrial construction. This highlights the TBiGNet model’s capability to maintain high accuracy while keeping computational costs under control.

Considering that lightweight models require less parameters and computation, they are not comparable with models with large computation, so they are only compared with models that provide computation and parameters. Table 5 shows that our model has the best effect in the overall RMSE and Score, indicating that our method delivers the most effective results overall. The best results in Table 5 have been presented in bold.

**Figure 11:**
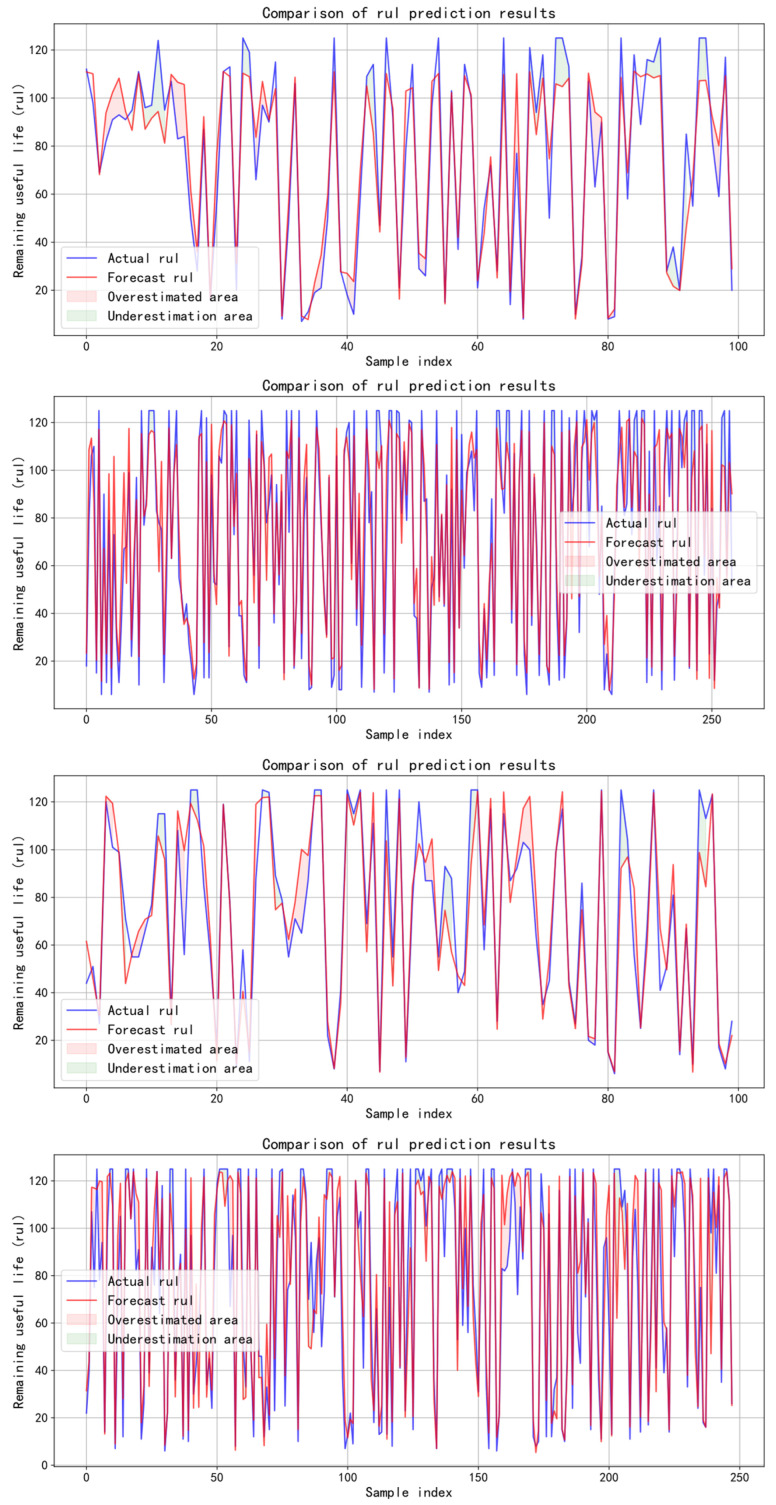
RUL prediction results of four sub datasets.

The prediction results of four sub datasets through the model are shown in Figure 11. When the data becomes more complex, RMSE still performs best. Because the data complexity of FD001 and FD003 is relatively low, some models have specially optimized them, so good results have been achieved on these sub datasets. However, this strategy has insufficient generalization ability on FD002 and FD004, which have more complex structures, leading to performance degradation. At the same time, some studies have tried to uniformly optimize all four sub datasets. Although this method helps to improve the overall performance, it usually brings high computing overhead. By comparison, the proposed TBiGNet model demonstrates strong generalization performance.

**Figure 12:**
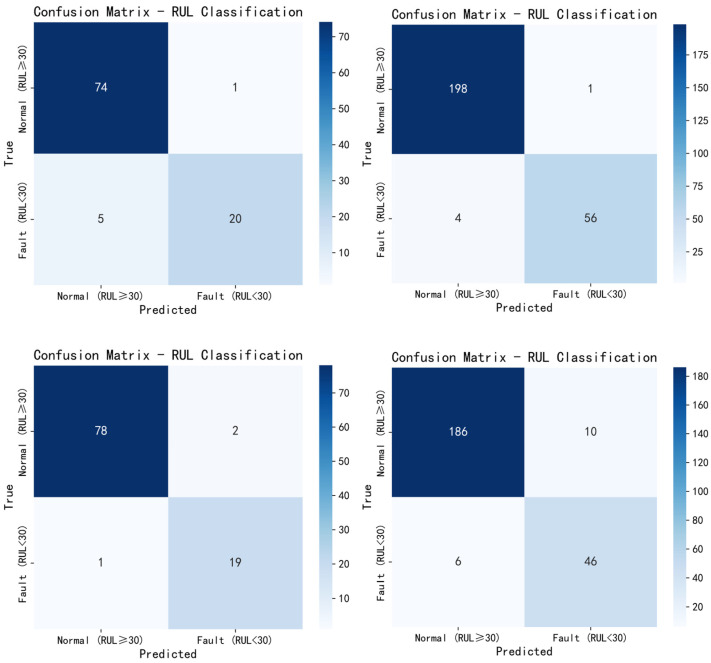
Classification prediction accuracy confusion matrix of four sub datasets.

In Figure 12, the overall accuracy of equipment operation state prediction of FD001 test set is 94.00%, FD002 98.07%, FD003 97.00%, and FD004 93.55%. The internal numbers in the figure are the samples of four test sets. There are 100 test samples in FD001, 259 in FD002, 100 in FD003, and 249 in FD004. Figure 10 shows that the simple threshold classification method is very effective and achieves high accuracy. In practical application, this classification result is helpful for people to judge the current machinery operation situation, and the staff will make corresponding decisions according to the current machinery operation status to prevent the occurrence of serious accidents in the future.

To assess its performance, the TBiGNet model was rigorously tested on each of the four sub-datasets within the C-MAPSS dataset. On the basis of Transformer, the model uses an efficient multi head attention module, an adaptive clipping module, and a feature fusion decoder to effectively deal with the complex timing problem of engine degradation prediction. At last, the threshold classification method is used to complete the classification of the current mechanical state.

### 4.4. Ablation Experiment

Ablation experiments were performed across the four data subsets to assess the contribution of each internal module: the efficient multi-head attention, adaptive clipping, and feature fusion decoder. The Transformer is a traditional transformer model with two layers of encoder and decoder stacked. Model 1 is designed to change the traditional Transformer encoder module into the encoder module with efficient attention mechanism and adaptive feature clipping layer designed in this paper, in which the encoder and decoder are also stacked with two layers. Model 2 is to change the traditional Transformer decoder module into the decoder module with BiGRU feature fusion in this paper, in which only the decoder has two layers stacked. TBiGNet is the complete model proposed in this paper. Based on two variant models and a traditional Transformer model, experiments were conducted on four subsets. To assess the model performance, RMSE and Score are adopted as evaluation indicators. The accuracy results are summarized in Table 6, and the model efficiency in terms of computation and parameters is shown in Table 7. The best results in Table 6 and Table 7 are shown in bold.

### 4.5. Complexity Analysis

The magnitude of computational workload directly affects the time complexity of algorithm execution, determining the speed of model training or inference. The memory access volume refers to the total amount of memory exchange that occurs during the forward propagation process of a model for a single input sample, which also represents the spatial complexity of the model. The parameter count denotes the total number of parameters in the model, which is directly related to the storage space required on the disk.

As demonstrated in Table 7, the experimental results of Model 1 confirm that the Efficient Multi-head Attention and Feature Pruning Layer effectively enhance model performance. Compared with the traditional Transformer model, Model 1 achieves lower RMSE and Score values across four sub-datasets, indicating that the Efficient Multi-head Attention and Feature Pruning modules excel at extracting relatively important features. Notably, the computational workload, parameter count, and memory access volume are reduced by nearly 50% compared to the encoder in the Transformer model.

In the Model 2 experiment, the decoder outperforms the traditional Transformer decoder in both prediction accuracy and computational efficiency, with significant reductions in computational workload, parameter count, and memory access volume. This demonstrates that the designed decoder effectively fuses features for decoding and prediction.

As compared to Model 2, Model 1 demonstrates superior performance in handling simple datasets, while Model 2 exhibits better effectiveness in processing more complex datasets (FD002 and FD004). This further confirms that the multi-scale feature fusion of the decoder can effectively address complex problems. Specifically, Model 1 employs a Feature Pruning module, which may inadvertently prune relatively important features during the pruning process, leading to inaccurate predictions of Remaining Useful Life (RUL). The more complex the dataset, the greater the number of omitted features, which explains why Model 1 only performs well on simple datasets but shows unstable performance on complex ones. In contrast, the feature fusion in Model 2 enhances model stability, effectively addressing the limitations of Model 1.

Notably, both Model 1 and Model 2 outperform the traditional Transformer model in terms of reduced computational workload and parameter count, while achieving improved prediction accuracy.

The proposed TBiGNet model demonstrates significant improvements over the traditional Transformer model in both accuracy and efficiency. Specifically, the mean RMSE is reduced by over 15%, the mean Score decreases by more than 60%, and the computational workload, parameter count, and memory access volume are all reduced by over 98%.

In terms of structural design, the encoder with an Efficient Multi-head Attention mechanism and an Adaptive Feature Pruning module reduces the parameter count, computational workload, and memory access volume compared to the traditional encoder. Additionally, the decoder with a feature fusion processing module preserves information from both original and pruned features, enhancing the model’s computational efficiency and accuracy in complex scenarios while improving its ability to capture multi-scale features.

Based on these improvements, experiments demonstrate that the proposed model outperforms the traditional Transformer model in both time and spatial complexity. Moreover, the lower computational workload of our model compared to existing models indirectly confirms that its computational complexity is lower than those proposed in other studies.

## 5. Conclusions

This paper presents TBiGNet, an efficient edge-deployable model for RUL estimation and classification, constructed using a lightweight Transformer architecture with multi-layer encoding and decoding components. The model effectively captures multi-scale timing features in the process of device degradation through the efficient multi head attention mechanism and feature clipping module in the encoder, and innovatively combines the feature fusion processing mechanism of original features and clipping features in the decoder. The outstanding performance of TBiGNet in remaining useful life prediction and fault classification tasks can be attributed to the encoder’s efficient multi-head attention and adaptive clipping mechanisms, as well as the feature fusion strategy employed in the decoder, all of which contribute to enhanced prediction accuracy. Model analysis shows that the method balances accuracy and efficiency well, allowing for efficient execution on edge devices with limited computing resources and providing more reliable decision support for the practice of intelligent industry. In the actual industrial environment, we may face problems such as industrial noise and data loss. Therefore, in future work, we will further optimize the feature clipping strategy, explore a more effective multi task learning framework, make the model more suitable for the real industrial environment, and expand the model to more complex industrial scenes and edge devices.

## Figures and Tables

**Table 1:** Data description of data subset [24].

#	Attribute	Description	Unit
1	ID	engine IDs	-
2	cycle	flight cycles	-
3	altitude	flight height of the aircraft	foot
4	Mach number	ratio of flight speed to speed of sound	-
5	sea-level temperature	flight temperature of the aircraft	°F
6	T2	total temperature at fan inlet	°R
7	T24	total temperature at LPC outlet	°R
8	T30	total temperature at HPC outlet	°R
9	T50	total temperature at LPT outlet	°R
10	P2	total pressure at fan inlet	psia
11	P15	total pressure in the bypass	psia
12	P30	total pressure at HPC outlet	psia
13	Nf	physical speed of low-pressure shaft	rpm
14	Nc	physical speed of high-pressure shaft	rpm
15	epr	engine pressure ratio	-
16	Ps30	static pressure at HPC outlet	psia
17	phi	ratio of fuel flow to Ps30	pps/psi
18	NRf	corrected fan speed of low-pressure shaft	rpm
19	NRc	corrected core speed of high-pressure shaft	rpm
20	BPR	bypass ratio	-
21	farB	burner fuel-air ratio	-
22	htBleed	enthalpy of bleed	-
23	Nf_dmd	demanded fan speed	rpm
24	PCNfR_dmd	demanded corrected fan speed	rpm
25	W31	HPT coolant bleed	lbm/s
26	W32	LPT coolant bleed	lbm/s

**Table 2:** C-MAPSS dataset information.

Dataset	FD001	FD002	FD003	FD004
Training set	100	260	100	249
Test set	100	259	100	248
Operating conditions	1	6	1	6
Fault status	1	1	2	2

**Table 3:** RMSE and score of various algorithms on C-MAPSS dataset.

Model	FD001		FD002		FD003		FD004	
	RMSE	Score	RMSE	Score	RMSE	Score	RMSE	Score
SVR	18.28	1004.75	30.50	17,132.17	21.37	2084.75	34.11	15,740.27
CNN	18.44	1286.70	30.29	13,570	19.81	1596.20	29.15	7886.40
DBN	15.21	417.59	27.12	9031.64	14.71	442.43	29.88	7954.51
ELM	17.27	523	37.28	498,149	18.90	573	38.43	121,414
GB	15.67	474.01	29.09	87,280.06	16.84	576.72	29.01	17,817.92
RF	17.91	479.75	29.59	70,456.86	20.27	711.13	31.12	46,567.63
LSTM-FNN	16.14	338	24.49	4450	16.18	852	28.17	5550
GAN	16.91	N/A	N/A	N/A	N/A	N/A	46.40	N/A
IDMFFN	12.18	**205**	18.19	10,412	11.89	**206**	21.72	3339
BIGRU-TSAM	12.56	213	18.94	2264	12.45	233	20.47	3610
AM-LSTM	14.53	322.44	N/A	N/A	N/A	N/A	27.08	5649.14
AGCNN	12.42	226	19.43	1492	13.39	227	21.50	3392
GCU-Transformer	**11.27**	N/A	22.81	N/A	**11.42**	N/A	24.86	N/A
Cau-AttnPINN	N/A	N/A	19.08	1665	N/A	N/A	20.70	3035
BTCAN	14.46	309	19.88	2800	12.79	298	22.03	4224
PAOLTransformer	12.49	257.71	21.63	1692.59	12.66	274.15	23.86	3163.41
**TBiGNet**	12.53	219.80	**13.67**	**812.10**	13.59	775.69	**17.40**	**2347.92**

**Table 4:** Comparison of calculation amount.

Model	Parameter Num	FLOPs
PAOLTransformer	2.6 × 10^5^	6 × 10^7^
GCU-Transformer	1,781,937	6.32 × 10^7^
AM-BGRU	18,629	1.58 × 10^6^
BIGRU-TSAM	2,825,443	1.68 × 10^8^
AM-LSTM	90,061	1.06 × 10^6^
**TBiGNet**	**1890**	**1.31 × 10^5^**

**Table 5:** Mean value of different models.

Model	RMSE Mean	Score Mean
PAOLTransformer	16.28	1346
GCU-Transformer	17.59	N/A
AM-BGRU	16.69	1334
BIGRU-TSAM	16.10	1580
AM-LSTM	20.81	2985
**TBiGNet**	**14.20**	**1038**

**Table 6:** Ablation experiment of model on CMPASS.

Model	FD001		FD002		FD003		FD004	
	RMSE	Score	RMSE	Score	RMSE	Score	RMSE	Score
Transformer	15.11	480.28	15.87	1780.14	15.33	607.16	20.68	6819.97
Model 1	14.22	425.51	15.48	1116.11	13.36	389.95	19.79	4822.15
Model 2	13.69	420.54	15.22	922.28	13.83	480.83	18.54	3181.45
**TBiGNet**	**12.53**	**219.80**	**13.67**	**812.10**	**13.33**	**370.81**	**17.40**	**2347.92**

**Table 7:** Efficiency analysis of the model.

Model	Parameter Num	Bytes	FLOPs
Transformer	1.39 × 10^5^	4.54 × 10^6^	7.89 × 10^6^
Model 1	7.18 × 10^4^	2.61 × 10^6^	4.09 × 10^6^
Model 2	7.03 × 10^4^	2.28 × 10^6^	3.98 × 10^6^
**TBiGNet**	**1890**	**2.72 × 10^5^**	**1.31 × 10^5^**

## Data Availability

https://github.com/huster123/c-mapss-full-dataset-/tree/master/Data (1 July 2025).
